# A pioneering genotypic and phylogenetic characterisation of *Cichorium* crops through a genome-scale sequencing for future breeding innovations

**DOI:** 10.1186/s12870-025-06876-1

**Published:** 2025-07-03

**Authors:** Francesco Scariolo, Samela Draga, Damiano Riommi, Alessandra Tondello, Lydia Grace Griffin, Fabio Palumbo, Alessandro Vannozzi, Gianni Barcaccia

**Affiliations:** 1 Department of Agronomy, Food, Natural Resources, Animals and Environment (DAFNAE), University of Padova, Agripolis, Legnaro, 35020 Italy; 2 Department of Crop & Soil Sciences, University of Georgia, 3111 Miller Plant Sciences Bldg., Athens, GA 30602 USA

**Keywords:** Chicory, Endive, Asteraceae, NGS, Genetic diversity

## Abstract

The genus *Cichorium*, which comprises economically important crops such as chicory and endive, exhibits significant genetic and phenotypic diversity. This study used genome-scale sequencing based on ddRAD technology to explore the genetic diversity and relationships, and to identify multiple discriminant loci within this genus. Moreover, microscopy analysis was conducted to identify morphological traits, such as pappus structure, to aid species-level identifications. Despite this, the genetic complexity within *Cichorium* remains challenging to resolve based solely on phenotypic characteristics. Our study confirms the efficacy of ddRADseq in generating high-quality genotyping-by-sequencing data, identifying 1,350 multi-allelic polymorphic loci across 368 genotypes and revealing significant genetic differentiation among species, varieties, and biotypes. Lower observed heterozygosity (Ho) compared to expected heterozygosity (Hs) suggests the presence of fixed genotypes within biotype groups, likely due to selection for specific morphological traits. Genetic distance and clustering analyses proved the distinctiveness of self-incompatible species (*C. intybus* and *C. spinosum*) from self-compatible species (*C. endivia* and *C. pumilum*). Notably, a strong genetic relationship was observed between *C. intybus* var. *foliosum* (Witloof) and var. *sativum* (Root chicory), supporting the hypothesis of a common ancestry. These findings may help refine the taxonomic classification of *Cichorium*, offering valuable insights for the conservation of genetic resources and their classification, the precise adoption in breeding decisions and genomic selection of suitable genotypes, as well as the genotypic identity determination of registered cultivars with the protection of breeder’s rights.

**Supplementary Information:**

The online version contains supplementary material available at 10.1186/s12870-025-06876-1.

## Core

This study revealed significant genetic diversity within the *Cichorium* genus, corroborating the taxonomic relationships between species and varieties, particularly *C. intybus*.

An advanced genome-scale sequencing analysis identified key genetic markers distinguishing species, biotypes, and botanical varieties, providing insights into their evolutionary history.

Our findings also suggest the potential for improved breeding strategies and cultivar development, which could have implications for conservation of genetic resources and genetic traceability of food products.

## Introduction

The genus *Cichorium*, which is part of the Asteraceae family, includes cultivated and wild species characterised by high genetic diversity [1–4]. The two most widely cultivated species, *Cichorium intybus* L. and *Cichorium endivia* L., have undergone extensive domestication and breeding, resulting in the development of numerous cultivars that are highly valued, either for their leaves, which are commonly used as salad greens or for their taproots, which are primarily used for inulin production and food consumption as a coffee substitute [5–7]. Although less widely cultivated for food production than their domesticated counterparts, wild species within the genus, mainly adopted in biodiversity studies and ornamental destinations, hold valuable genetic backgrounds for breeding programs, contributing to the genetic variability of the *Cichorium* genus. These wild species include *C. bottae* Deflers, *C. spinosum* L., *C. calvum* Sch. Bip., and *C. pumilum* Jacq. The main morphological traits adopted in the taxonomic classification of species within *Cichorium* have been the shape and colour of leaves and flowers. However, an intriguing additional trait has been observed: the pappus structure, a distinctive feature of chicory florets that is believed to be homologous to sepals and plays a pivotal role in the classification within the Asteraceae family [2, 8]. The pappus structure in *C. endivia* and C. *pumilum* is distinguished by large scales, with subtle variations observed between the two species. In contrast, *C. calvum* exhibits the most pronounced divergence from the other *Cichorium* species, characterised by a pappus comprising of minute scales, as observed for the first time by Kiers et al. [9], whereas *C. intybus* has a relatively small-scaled pappus but is more pronounced than *C. calvum* is. Nevertheless, overall characteristics, such as the typical cushion-like growth form exhibited by *C. bottae*, the presence of spiny terminal branches in *C. spinosum*, or the almost non-existent pappus in *C. calvum*, could undoubtedly contribute to the classification between species within *Cichorium* [9, 10]. Further studies are needed to address the phylogenetic relationships between *C. intybus* and *C. spinosum*, given the challenges encountered in genetically distinguishing them, which are likely attributed to an early stage in the speciation process [4, 10].

The genetic structure of wild and cultivated chicory species is weak and limited, with different taxonomic classifications proposed by different researchers over the years [2, 4, 9, 10]. Thus, employing molecular data and computational methods, rather than relying solely on traditional morphology-based systems, is pivotal for revealing the taxonomic relationships within the *Cichorium* genus and reconstructing its evolutionary pathways and phylogenies at the molecular level. In recent decades, phylogenetic studies on *Cichorium* have utilised various molecular markers, including nuclear ribosomal DNA (rDNA), chloroplast DNA (cpDNA) sequences, nuclear ribosomal internal transcribed spacer (ITS), random amplified polymorphic DNA (RAPD), amplified fragment length polymorphisms (AFLPs), simple sequence repeats (SSRs), expressed sequence tags (ESTs), and single-nucleotide polymorphism (SNP) markers, to elucidate the genetic relationships within *Cichorium* [4, 9–11]. The results of these studies demonstrated that *C. bottae* can be easily distinguished from the other *Cichorium* species, while the remaining species can be classified into two main groups. One of these includes *C. intybus* and *C. spinosum*, two allogamous self-incompatible and perennial species, whereas the other comprises *C. endivia*, *C. pumilum*, and *C. calvum*, three annual autogamous and completely self-compatible species [9, 12, 13].

In particular, the intraspecific classification system of *C. intybus* has attracted great interest. It includes, according to the classification from Barcaccia et al. [13], five main botanical varieties that represent different cultivated biotypes from this species: *i*) var. *latifolium* (Italian Radicchio), *ii*) var. *foliosum* (Belgian endive), *iii*) var. *porphyreum* (Pain de Sucre), *iv*) var. *sylvestre* (Catalogne) and *v*) var. *sativum* (Root chicory).

In *C. endivia*, the taxonomic complexity is lower, with two main typologies reported: curly and smooth endives. These two types are distinguishable mainly by the shape of their green leaves, which are curly in *C. endivia* var. *crispum* (Endive) and smooth in *C. endivia* var. *latifolium* (Escarole). Endives are often used in upscale dining and salads because of their slightly bitter flavour and crisp texture and are cooked in many traditional dishes from Mediterranean regions [14, 15].

Among the botanical varieties specific for *Cichorium intybus*, var. *foliosum* is especially notable for its significant agricultural and economic impact. Widely appreciated for its leaves, which are consumed raw or cooked and characterised by a distinctive bitter taste and crisp texture, this botanical variety includes the well-known ‘Belgian endive’, or ‘Witloof chicory’, biotype. ‘Belgian endive’ is cultivated for its etiolated buds, known as ‘chicons’, through a meticulous process involving the harvesting, storage, and forced darkness of chicory roots to promote the characteristic pale colour and delicate texture [16]. This variety is highly regarded in European cuisine, especially in Belgium, the Netherlands, and France, for its mild bitterness and crispness [17].

*C. intybus* var. *sativum*, which represents the typology named ‘Root chicory’, is cultivated primarily for its taproot. ‘Root chicory’ plays a crucial role in the agricultural and industrial sectors because of its economic importance and versatile applications in the food and nonfood industries, including inulin production [1, 18, 19].

Multiple typologies are present in the Italian cultivation of *C. intybus*, which are distributed in different areas according to specific environmental needs and traditional uses. An example is that of the “radicchio” types, which are mainly distributed in northeastern Italy; the general common name “radicchio” includes multiple local biotypes that share the presence of red-coloured leaves (completely or variegated) but differ in morphology, genetics and area of cultivation. Radicchio biotypes are named after their places of origin (e.g., ‘red of Treviso’, ‘red of Chioggia’, ‘red of Verona’, and ‘variegated of Castelfranco’), and they are differentiated by presenting specific traits and phenotypes resulting from decades or centuries of local selection [13, 20, 21]. Historically, phenotypic mass selection has been the primary method for developing uniform and high-yielding radicchio populations, which led to the differentiation of local selections, which are now considered specific biotypes. Modern breeding strategies in radicchio, on the other hand, have evolved from phenotypic mass selection to genotypic selection of the best individuals assisted by molecular markers and genomic sequencing approaches [21–23]. Renowned for its adaptability to diverse environmental conditions, radicchio is widely cultivated and holds significant economic value, particularly in the European market.

Var. *porphyreum* and var. *sylvestre* represent two typical Italian biotypes. Var. *porphyreum* has the so-called “Pain de Sucre” typology, which presents long green-coloured leaves and low bitterness. Var. *sylvestre* has the “Catalogne” typology represented by the “Catalogna” and “Puntarelle” biotypes, which are typically cultivated in the central-southern regions of Italy and are characterised by green leaves and are cultivated for their stems.

Crops and wild species within the *Cichorium* genus have been studied for different purposes related to phylogeny and breeding, utilizing molecular tools such as PCR-based molecular markers or Next Generation Sequencing (NGS) strategies. However, many of these studies suffer from limited representativeness, often focusing exclusively on wild accessions, subsets of the discussed taxa, or specific typologies and cultivars within this genus [22, 24–26]. As a result, the taxonomy and phylogeny of *Cichorium* remain unclear, particularly at the intraspecific level.

To address this issue and following the taxonomic classification proposed by Barcaccia et al. [13] and described above, we conducted a comprehensive analysis of 42 cultivated and pre-commercial varieties of *C. intybus*, which are representative of its different biotypes and botanical varieties, along with 8 cultivated varieties of *C. endivia* and three wild species accessions. The analysis was conducted using double-digested Restriction-site Associated DNA sequencing (ddRADseq), a method widely adopted in studies related to plant phylogeny and genotyping [27–30]. In addition, we observed chicory florets under a microscope to investigate the putative presence of other morphological traits that can discriminate *Cichorium* species from a morphological point of view and to further confirm the previously observed morphologies of the pappus, as reported in the literature [2, 8].

This study aimed to reconstruct a reliable and useful phylogenetic representation of this genus and to provide new insights into the discriminant genomic region and morphological traits of florets, which could be valuable for future research on these species. The overarching objective was to investigate and elucidate the genetic diversity within and among species, represented in this study by over 300 samples overall, with particular focus on *C. intybus* var. *latifolium* different biotypes. Phylogenetic analyses continue to provide valuable insights into the genetic relationships and evolutionary history of chicory and its relatives by enhancing the understanding of the genomic interactions occurring between the taxonomic groups of this genus. Advancements in genomic and bioinformatics tools in recent years have produced a large amount of data regarding mapped markers and genomic linkage maps saturated with DNA polymorphic regions for chicory, thus contributing to its phylogeny and the exploitation of innovative genomic-based systems for applicative purposes such as breeding and traceability [24, 31]. Indeed, multiple molecular insights have been provided by genetic and genomic studies conducted worldwide on these species. The utilisation of molecular tools in genotyping elite breeding stocks of chicory has been pivotal in the assessment of genetic characteristics at the individual plant, breeding stock, and lineage levels [22, 24–26], but the need for a more comprehensive and broad-range taxonomic overview of this genus remains.

The results obtained in the present study aim to address these gaps of knowledge by providing new and valuable information about discriminant genomic regions at the species, varietal and cultivar levels. Furthermore, these findings could pave the way for developing innovative targeted genotyping strategies for *Cichorium* species. Such strategies could serve both basic and applied research purposes, including genomic-assisted breeding (GAB) and distinctiveness, uniformity and stability (DUS) testing, as well as molecular traceability and plant variety protection (PVP) such as approaches against fraud and illicit commercialisation of registered *Cichorium* spp. cultivars.

## Materials and methods

### Structural visualisation by aniline blue staining

Florets from opened flowers of *C. intybus* var. *latifolium*,* C. intybus* var. *sativum*, *C. endivia* var. *crispum*, *C. pumilum* and *C. calvum* were collected. Florets were carefully isolated from the capitula, and the ligule (the structure analogous to the petals in Asteraceae) was removed for analysis simplification. The florets were fixed in a mixture of acetic acid and absolute ethanol (1:3), washed three times with water, cleared with 8 N sodium hydroxide overnight, and labelled with aniline blue after all the sodium hydroxide was removed via several washes with water. Microscopic observations were conducted using a Nikon eclipse Ts2R microscope (Nikon, Tokyo, Japan) with inverted fluorescence. Whole pictures of the florets were acquired via manual large-capturing method.

### Plant material and genomic DNA extraction

In this study, 403 individuals belonging to *C. intybus*, *C. endivia*, *C. spinosum*, *C. pumilum* and *C. calvum* were considered. *C. intybus* was represented by five main botanical varieties, namely, var. *latifolium*, var. *foliosum*, var. *porphyreum*, var. *sylvestre* and var. *sativum*, comprising seven, one, one, two and one biotypes, respectively. In particular, var. *sativum* and var. *foliosum*, represented by root chicory (Root) and Belgian endive (BelgEnd) typologies, were selected together with var. *latifolium*, represented by 7 biotypes (namaley Late red of Treviso - TvT, Early red of Treviso - TvP, red of Verona - Vr, Variegated of Castelfranco - CF, Variegated of Chioggia - ChV, red of Chioggia - ChR, white of Chioggia - ChW), var. *porphyreum* represented by Pain de Sucre, or “Pan di Zucchero” (PanZ), and var. *sylvestre*, comprising both Catalogna (Cat) and Puntarelle (Punt) biotypes. Regarding endives, two biotypes were considered, named “Indivia Liscia” (EnL) and “Indivia Riccia” (EnR), belonging to var. *latifolium* and var. *crispum*, respectively.

Overall, biotypes from *C. intybus* var. *latifolium*, var. *porphyreum*, var. *sylvestre*, and *C. endivia* var. *crispum* and *latifolium* were represented by four different populations each, whereas the *C. intybus* var. *sativum* and var. *foliosum* biotypes were represented by one population each. Each population consisted of eight samples. *C. spinosum* (Cspin), *C. calvum* (Ccal) and *C. pumilum* (Cpum) consisted of one sample per species. Samples from *C. intybus* var. *latifolium*, var. *porphyreum*, and var. *sylvestre*, as well as *C. endivia*, were kindly provided by Blumen Group S.p.A. (Milan, Italy), whereas *C. intybus* var. *sativum* and var. *foliosum* accessions were purchased from Fratelli Ingegnoli S.p.A. (Milan, Italy). Accessions of *C. calvum* (PI 652029) and *C. pumilum* (PI 652032) were kindly donated by the United States Department of Agriculture - Agricultural Research Service, North Central Regional Plant Introduction Unit (Ames, IA, USA), and *Cichorium spinosum* plants were purchased from the private seller Pepiniere botanique de Vaugines (Vaugines, France). The plant material summary is reported below in Table 1, which presents the common names of the biotypes, their ID codes and the respective species, whereas the complete plant material description is reported in Supplementary Table 1, which is enriched with hierarchical clustering and divides the samples by population, biotype, typology and species.

**Table 1:** Sample list highlighting the taxonomic division of the core collection according to Barcaccia et al. [13]. Groups, species, botanical varieties (Var) and biotypes are indicated for each population. The number (n) of populations and samples are also reported for each group

Group	Species	Amphimictic strategy	Var	Biotype	Populations (*n*)	samples (*n*)
***Overall***	*5*		*7*	14	53	403
Wild					3	3
	*C. calvum*	*Autogamous*			1	1
	*C. pumilum*	*Autogamous*			1	1
	*C. spinosum*	*Allogamous*			1	1
Endive					8	64
	*C. endivia*	*Autogamous*				
			*crispum*	Curly Endive (EnR)	4	32
			*foliosum*	Smooth Endive (EnL)	4	32
Chicory					42	336
	*C. intybus*	*Allogamous*				
			*sativum*	Root Chicory (Root)	1	8
			*porphyreum*	Pain de Sucre (PanZ)	4	32
			*foliosum*	Belgian Endive (BelgEnd)	1	8
			*sylvestre*		8	64
				Catalogna (Cat)	4	32
				Puntarelle (Punt)	4	32
			*latifolium*		28	224
				Late Red of Treviso (TvT)	4	32
				Early Red of Treviso (TvP)	4	32
				Red of Verona (Vr)	4	32
				Red of Chioggia (ChR)	4	32
				Variegated of Castelfranco (CF)	4	32
				Variegated of Chioggia (ChV)	4	32
				White of Chioggia (ChW)	4	32

Genomic DNA (gDNA) extraction was conducted from young leaves using the DNeasy Plant 96 Kit and the DNeasy Plant Mini Kit (Qiagen, Hilden, Germany) based on the number of samples and their organisation in the sampling plates or tubes, respectively. gDNA was extracted following the protocols provided by the supplier and evaluated with a Qubit Flex Fluorometer (Thermo Fisher Scientific, Waltham, MA, USA) using the Qubit™ 1× dsDNA HS Assay Kit from the same manufacturer.

### DdRAD library preparation

The genomic DNA from each sample was normalised, and a total of 200 ng per sample was used for library preparation. The protocol of Abed et al. [32] was used for ddRAD library preparation with some modifications. The gDNA was double-digested with PstI (1 µl of FastDigest PstI per reaction, Thermo Fisher Scientific) and MspI (1 µl of FastDigest MspI per reaction, Thermo Fisher Scientific) restriction enzymes at 37 °C for 2 h, following the provided digestion protocols. Oligonucleotides were designed and prepared to formulate common and barcoded adapters as described by Abed et al. [32]. Barcode adapter and common adapter ligation to the restricted samples was carried out in a total volume of 50 µl per reaction containing 30 µl of the restricted DNA, 5 µl of the barcoded adapters (0.1µM) and common adapters (10µM), 1 µl of the T4 DNA ligase and 5 µl of a 10X T4 ligation buffer (T4 DNA Ligase, New England Biolabs) for 2 h, followed by a heat-inactivation step at 65 °C for 20 min.

The 403 digested and ligated samples were divided into pools, and a purification step was performed with the QiAquick PCR and Gel Cleanup Kit (Qiagen, Hilden, Germany) to remove unligated adapters, dimers and other small fragments and undesired impurities (proteins, enzymes, buffers, etc.). Library size selection from 100 bp to 600 bp was performed with magnetic bead CleanNGS (CleanNA, The Netherlands). After size selection, pooled libraries were amplified using Platinum SuperFi PCR Master Mix (Thermo Fisher Scientific) following the enrichment PCR protocol of Abed et al. [32]. The amplified libraries were further purified with magnetic beads CleanNGS (CleanNA) and quantified using the TapeStation System with High Sensitivity D1000 DNA ScreenTape assays (Agilent Technologies, Santa Clara, CA, USA). Libraries were then diluted to 100 pM before the following steps.

### Template preparation, chip loading, and ion Torrent sequencing

Template preparation and chip loading of the 100 pM diluted libraries were performed with the Ion Chef instrument overnight and using Chip 550 or Chip 530 (Thermo Fisher Scientific) according to the number of samples to be sequenced (~ 12 libraries/Chip 530; ~48 libraries/Chip 550). The template preparation and chip loading were automatised in the Ion Chef instrument following the default “200 bp” protocol implemented with in-house parameters. After the libraries were loaded on chips, the samples were sequenced on the Ion GeneStudio™ S5 System (Thermo Fisher Scientific) following the manufacturer’s instructions, depending on the adopted sequencing support (Chip 530 or Chip 550). Templates were sequenced with the read length set at 200 bps and the number of flows at 500 to perform two chips sequencing a day with one sequencer initialisation.

After sequencing, all the obtained reads from each sample were demultiplexed, quality-checked, filtered and mapped to the reference genome of *C. intybus* (GeneBank assembly: GCA_023525715.1) by the pipeline implemented in the Torrent Suite platform of the sequencer, following the default setting. Specifically, the default parameters for base-calling runs on Chip 530 were “BaseCaller --trim-qual-cutoff 15 --barcode-filter-minreads 10 --phasing-residual-filter = 2.0 --num-unfiltered 1000 --barcode-filter-postpone 1 --qual-filter true --qual-filter-slope 0.040 --qual-filter-offset 1.0 --wells-normalisation on”, whereas the default parameters for base-calling runs on Chip 550 were “BaseCaller --trim-qual-cutoff 15 --barcode-filter-minreads 10 --phasing-residual-filter = 2.0 --max-phasing-levels 2 --num-unfiltered 1000 --barcode-filter-postpone 1”. After the reads were mapped to the reference genome of *C. intybus*, BAM files were obtained for each sample and then retrieved from the local workstation using the *FileExporter* plugin implemented in the Torrent Suite platform. The retrieved data were then used in the bioinformatic analyses.

### Variant calling and quality control procedures

Variant calling was performed using Stacks2 software [33]. Through the command *gstacks* of the Stacks2 pipeline, BAM files were analysed with the following parameters: model = marukihigh, var-alpha = 0.10, and gt-alpha = 0.10. Phasing was not possible, and PE was not available because of the protocol adopted for library preparation. After the *gstacks* command, the *population* command was used with the parameters *r* = 0.30 and *R* = 0.65 to restrict the selection of variants and loci to those conserved in at least 30% of the samples belonging to the same population and 65% overall. The final VCF file used included only multi-allelic haplotypes reconstructed for each locus from the *catalog* derived from Stacks software, rather than individual SNPs or indels. The haplotypes, along with the corresponding genotypes for each of the 368 individuals, were retained as the final dataset for downstream population genetic analyses. This approach was chosen to capture the full allelic diversity within each locus, providing a more comprehensive representation of genetic variation than single-marker datasets.

A subsequent multistep variant and sample filtering process was conducted using VCFtools v0.1.17 [34]. Initially, variants with more than 20% missing data were removed from the VCF file of the haplotypes by applying a filter to retain only those variants with at least 80% of genotype calls present across all samples. Next, samples with more than 20% missing data were excluded from the dataset. This resulted in the removal of 35 samples, which were distributed across different populations with a number of samples discarded per population ranging from one to a maximum of four (e.g. EnL4). Finally, a minor allele frequency (MAF) filter was applied to retain only variants with an MAF threshold of 0.05.

### Statistics for population genetic diversity and differentiation

The filtered VCF was imported into RStudio software (https://posit.co/products/open-source/rstudio/) as a vcfR object, using the vcfR package [35, 36]. Then, a *genind* object, used as input for further analyses, was obtained from the vcfR object using the *adegenet* package [37, 38]. The *genind* object was also enriched with population and hierarchical clustering information to allow comparisons at different levels (Table S1).

Genetic statistics were computed using the *hierfstat* package [39]. First, the *basic.stat* command was used to compute the mean observed (Ho) and expected heterozygosity (Hs) within each group at each hierarchical order, the expected overall gene diversity (Ht) and the fixation index (Fst), and then, the Weir and Cockerham pairwise Fst was computed in all pairwise comparisons using the *pairwise.WCfst* command and plotted as heatmaps and matrices to highlight the distinctiveness among the considered hierarchical orders of varieties, biotypes, types and species. Observed heterozygosity, computed as averages in 1Mbp genomic windows for the two most represented species in the dataset (*C. intybus* and *C.* endivia) were further plotted using the ChromPlot package version 1.32.0 [40, 41]. The plot was created using different colours to represent the number of polymorphic loci identified in each window (represented as bars within the chromosomic map), while average Ho for the two species were plotted as coloured dots. Finally, analyses of molecular variance (AMOVAs) were conducted using the *poppr.amova* command, and considering biotypes/populations orders, species/types orders and species/types/biotypes/population orders. The obtained results were validated using the *randtest* function from the *ade4* package to check the significance of the computed molecular variances at different levels with 999 permutations.

### Genetic structure analysis

The *aboot* command from the *poppr* package [42] was used to create a UPGMA dendrogram based on Nei’s genetic distance [43], with 1000 bootstrap replicates, and then plotted using the *plot.phylo* command from the *ape* package [44, 45]. Nei’s genetic distances among the selected hierarchical orders were also computed using the *dist.genpop* command from *adegenet*, and the resulting matrix was organised into tables and heatmap figures. Additionally, principal component analysis was performed using the *dudi.pca* command from the *ade4* package. The PCA graphical representation was plotted using the ggplot2 package [46].

To further investigate the genetic structure of the core collection, the dataset obtained from the filtered VCF file was converted into an input file for STRUCTURE software and used to determine the most likely number of K, corresponding to the putative number of ancestral populations into which our samples could be divided. The genotypic dataset was analysed with several K values between 1 and 60, using 5 replicates for each K with 2*10^5^ burn-in iterations and 10^6^ MCMC (Makarov Chain Monte Carlo) steps. Following Evanno et al. [47], the most likely values of K were evaluated through STRUCTURE HARVESTER software [48–50], and the obtained results were organised and plotted as barplot using an Excel spreadsheet.

A maximum likelihood dendrogram was also constructed to infer the genetic relationships between the samples. The analysis of the filtered dataset was conducted using iQTree2 software [51]. The best model of allelic substitution and across-site heterogeneity in evolutionary rates (GTR2 + F0 + R10) was inferred using ModelFinder [52] based on the Bayesian information criterion (BIC). Supports to nodes and branches were also computed via Ultra-Fast Bootstrap (UFB) [53] and the Shimodaira–Hasegawa approximate likelihood ratio test (SH-aLRT) [54], with 10,000 replicates each. The dendrogram was then plotted using FigTree v1.4.4 software (https://github.com/rambaut/figtree), and the accession names were coloured to better highlight them in the graphics.

## Results

### Microscopic observations

**Fig. 1:**
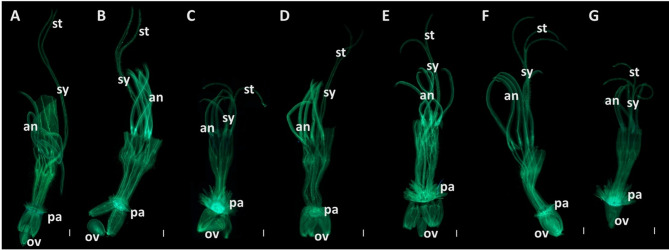
Florets of seven different *Cichorium* samples. Panels **A–E** species with typical bifid stigmas — **A** *C. intybus* var. *sativum*, **B** *C. intybus* var. *latifolium*, **C** *C. endivia* var. *crispum*, **D** *C. calvum*, and **E** *C. pumilum*. Panels **F–G** cultivated species with trifid stigmas — **F** *C. intybus* var. *latifolium*, and **G** *C. endivia* var. *crispum*. The ligule was dissected before fixation. Abbreviations: *st*, stigma; *sy*, style; *an*, anthers; p.a., pappus; *ov*, ovary. Scale bars: 250 μm

Chicory florets were subjected to microscopic examination to characterise the morphological differences among different species. The initial step involved examining the entire florets of four species and two botanical varieties, *C. intybys* var. *latifolium*, *C. intybus* var. *sativum*, *C. endivia* var. *crispum*, *C. calvum* and *C. pumilum*, using aniline blue staining to observe their general structures. Bifid stigmas are typically observed in the *Cichorium* genus (Fig. 1A-E) and the Asteraceae family, except *Antennaria dioica*, where both bifid and trifid stigmas have been documented [55]. However, our observations revealed an unreported feature in the *Cichorium* genus: the occurrence of trifid stigmas in the cultivated species *C. intybus* var. *latifolium* and *C. endivia* var. *crispum* (Fig. 1F, G). Interestingly, florets with both bifid and trifid stigmas were observed simultaneously within the same inflorescence of these species, suggesting previously undocumented morphological variability.

**Fig. 2:**
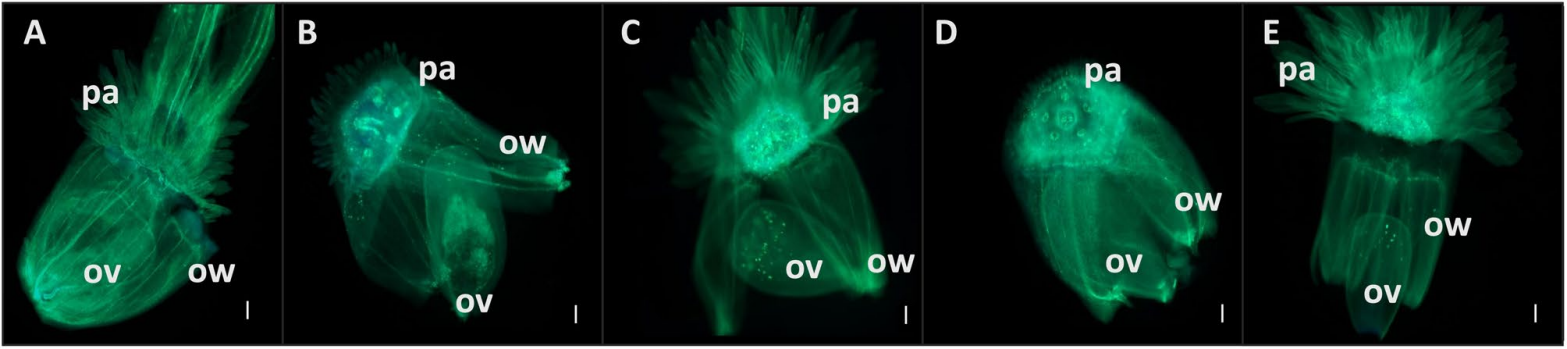
Microscopic observations of the ovaries and papillae of four different species of *Cichorium*: **A** *C. intybus* var. *sativum*, **B** *C. intybus* var. *latifolium*, **C** *C. endivia* var. *crispum*, **D** *C. calvum*, and **E** *C. pumilum*. p.a., pappus; *ov*, ovary; *ow*, ovary wall. Scale bar, 100 μm

Second, microscopic observations of the pappus structure (Fig. 2) yielded results consistent with those previously reported by Kiers [2]. The pappus structure is similar in size and shape between *C. endivia* (Fig. 2C) and *C. pumilum* (Fig. 2E), whereas *C. intybus* (Fig. 2A, B) presents a notably smaller pappus. However, it remains larger than *C. calvum* (Fig. 2D), which is morphologically distinguishable owing to its very minute-scale pappus.

A notable distinction was observed between the two varieties of *C. intybus*, var. *sativum* and var. *latifolium*, with the pappus scales of var. *sativum* (Fig. 2A) being larger and distinguishable from those of var. *latifolium* (Fig. 2B).

### Sequencing and dataset filtering

Overall, ddRAD sequencing of the 403 *Cichorium* accessions considered in this study produced 1.03 billion reads with an average read length of 161.5 bp after raw data processing and an average of 2.55 million reads per sample. The overall number of sequenced bases was above 1.66 × 10^11^, with 84.1% of the bases achieving a quality score above Q20. These sequencing results indicate high data throughput based on the selected protocol. Table 2 summarises the results, divided by taxonomic group, reporting overall values and per-sample averages.

**Table 2:** Sequencing results of the 403 DdRADseq genotypes after raw read filtering and mapping. Overall (O) values and per-sample averages (A) are reported for the different groups

Group	Species	Botanical variety	Biotype	O/A	Bp (*n*)	≥Q20 Bp (*n*)	≥Q20 Bp (%)	Reads (*n*)	length (bp)
				O	1.66 × 10^11^	1.40 × 10^11^		1.03 × 10^9^	
				A	4.1 × 10^8^	3.5 × 10^8^	84.10%	2.6 × 10^6^	161.5
Wild	*-*			A	1.5 × 10^8^	1.4 × 10^8^	94.40%	7.9 × 10^5^	185.3
	*calvum*			A	6.6 × 10^7^	6.2 × 10^7^	95.00%	3.4 × 10^5^	191
	*pumilum*			A	2.6 × 10^8^	2.5 × 10^8^	95.10%	1.3 × 10^6^	191
	*spinosum*			A	1.2 × 10^8^	1.1 × 10^8^	93.00%	7.0 × 10^5^	174
Endive	*endivia*			A	4.4 × 10^8^	3.6 × 10^8^	81.10%	2.9 × 10^6^	154.9
		*crispum*	Curly Endive (EnR)	A	4.2 × 10^8^	3.4 × 10^8^	81.40%	2.7 × 10^6^	155
		*foliosum*	Smooth Endive (EnL)	A	4.7 × 10^8^	3.8 × 10^8^	80.80%	3.0 × 106	154.7
Chicory	*intybus*			A	4.1 × 10^8^	3.5 × 10^8^	84.60%	2.5 × 10^6^	162.5
		*sativum*	Root Chicory (Root)	A	3.5 × 10^8^	3.3 × 10^8^	92.10%	1.9 × 10^6^	187.8
		*porphyreum*	Pain de Sucre (PanZ)	A	4.7 × 10^8^	3.9 × 10^8^	82.40%	2.8 × 10^6^	167
		*foliosum*	Belgian Endive (BelgEnd)	A	4.6 × 10^8^	4.2 × 10^8^	92.30%	2.4 × 10^6^	187.3
		*sylvestre*		A	3.6 × 10^8^	3.0 × 10^8^	81.90%	2.1 × 10^6^	168.8
			Catalogna (Cat)	A	3.6 × 10^8^	2.9 × 10^8^	82.00%	2.1 × 10^6^	168.9
			Puntarelle (Punt)	A	3.7 × 10^8^	3.0 × 10^8^	81.90%	2.2 × 10^6^	168.7
		*latifolium*		A	4.2 × 10^8^	3.5 × 10^8^	85.20%	2.6 × 10^6^	158.3
			Late Red of Treviso (TvT)	A	5.2 × 10^8^	4.5 × 10^8^	87.70%	3.3 × 10^6^	155.5
			Early Red of Treviso (TvP)	A	3.9 × 10^8^	3.4 × 10^8^	87.70%	2.5 × 10^6^	154.8
			Red of Verona (Vr)	A	5.1 × 10^8^	4.4 × 10^8^	87.50%	3.3 × 10^6^	154.3
			Variegated of Castelfranco (CF)	A	2.7 × 10^8^	2.2 × 10^8^	81.50%	1.8 × 10^6^	150.8
			Variegated of Chioggia (ChV)	A	3.9 × 10^8^	3.2 × 10^8^	83.90%	2.3 × 10^6^	165.1
			Red of Chioggia (ChR)	A	2.7 × 10^8^	2.3 × 10^8^	84.00%	1.7 × 10^6^	163.8
			White of Chioggia (ChW)	A	5.6 × 10^8^	4.7 × 10^8^	83.90%	3.4 × 10^6^	163.7

The retrieved sequencing data were then analysed using the Stacks2 pipeline, which identified more than 200,000 nucleotide variants across more than 30,000 loci. After applying a filtering threshold of 20% missing data for loci and genotypes, a final dataset of 1350 polymorphic loci with 8139 haplotype variants was obtained from 368 samples, ensuring the representativeness of all the populations considered. The list of polymorphic loci from the molecular dataset is provided in Supplementary Table 2, which includes the genomic coordinates, the haplotypic variants identified after filtering, and the catalogue of mapped loci derived from the Stacks software. Additionally, Supplementary Data 1 contains the FASTA sequences of these loci as retrieved from Stacks.

### Statistics for population diversity and differentiation

The final dataset, containing 1350 multi-allelic polymorphic loci distributed across the *C. intybus* reference genome, was used to calculate, for each hierarchical level, statistics related to genetic diversity and genetic differentiation.

The results revealed Ho mean values ranging from 0.149 (species) to 0.170 (biotype), while Hs values ranged from 0.174 (populations) to 0.264 (species). The overall average heterozygosity (Ht) ranged between 0.489 (populations) and 0.580 (species), consistent with the presence of multiple taxa, the majority of which are allogamous. The fixation index (Fst) values demonstrated significant distinctiveness across all hierarchical levels, with values higher than 0.545 at the species level. These results reflect substantial genetic variability across the genus *Cichorium*, with a progressive reduction in diversity when moving from interspecific comparisons to comparisons within populations and biotype groups (Table 3).

**Table 3:** Genetic statistics reporting the average observed heterozygosity (Ho) and expected heterozygosity (Hs), the overall expected heterozygosity (Ht), the inbreeding coefficient (Fis) and the fixation index (Fst). Statistics are indicated for each hierarchical grouping of the samples

Hierarchy	Ho	Hs	Ht	Fst
Species	0.149	0.264	0.580	0.545
Botanical varieties	0.159	0.251	0.568	0.559
Biotypes	0.170	0.221	0.532	0.584
Populations	0.160	0.174	0.489	0.645

**Fig. 3:**
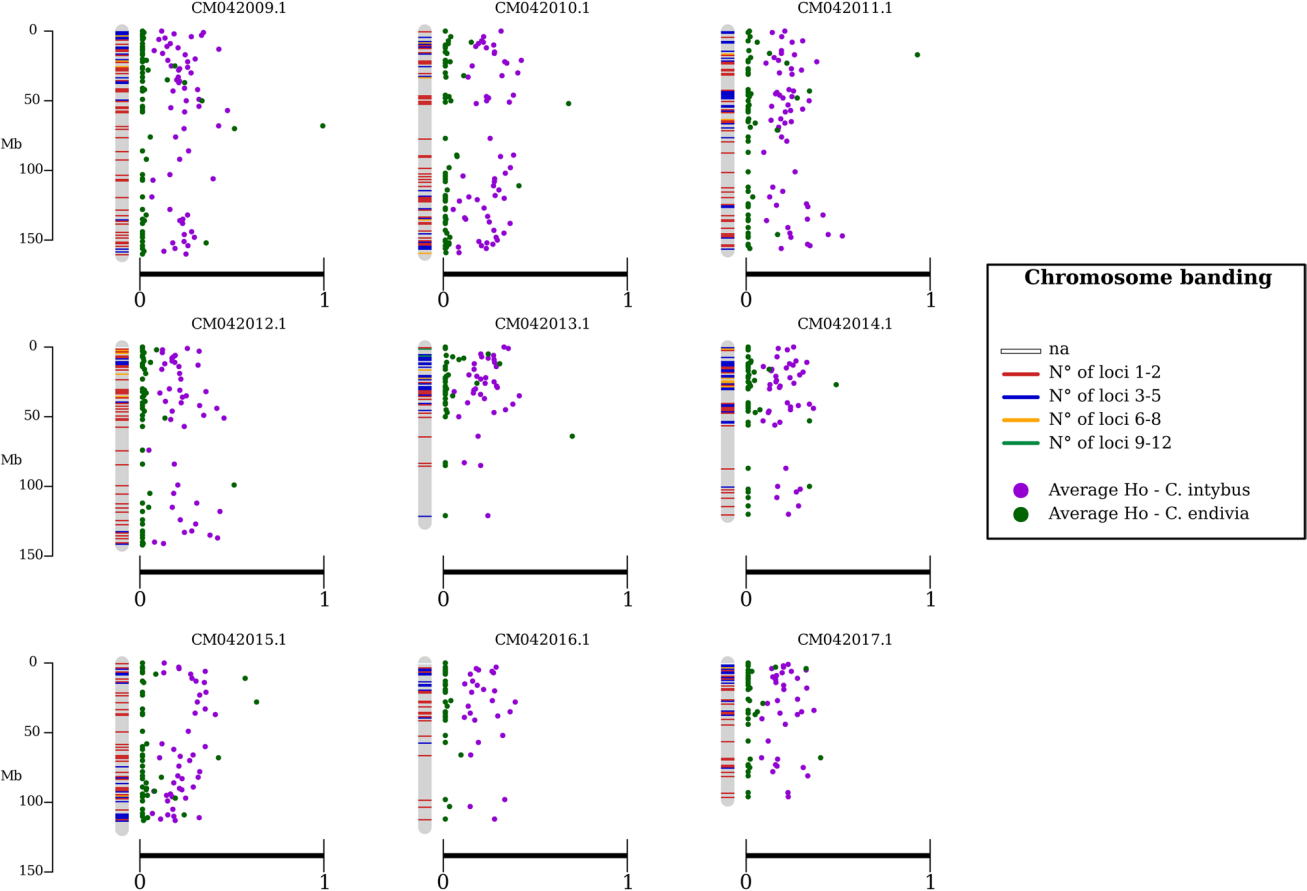
Chromosome map of *Cichorium intybus* showing average observed heterozygosity (Ho) values in 1 Mbp windows for *C. intybus* and *C. endivia*. Each chromosomal window is represented by a colored bar, categorized into four classes based on the number of polymorphic loci it contains (as indicated in the legend). Average Ho values are displayed as colored dots, with different colors used to distinguish between the two species

Results of the average Ho values for *C. intybus* and *C. endivia*, visualised as chromosomal maps in Fig. 3, revealed the distribution and heterozygosity of polymorphic loci. As shown in the maps, the loci retained in the final dataset were distributed across all nine chromosomes of the *C. intybus* reference genome, with the majority of chromosomic windows having higher heterozygosity in *C. intybus* rather than in *C. endivia*. The number of polymorphic loci per window ranged between 1 and 12, although the majority of which presented 1 to 5 polymorphic loci.

**Fig. 4:**
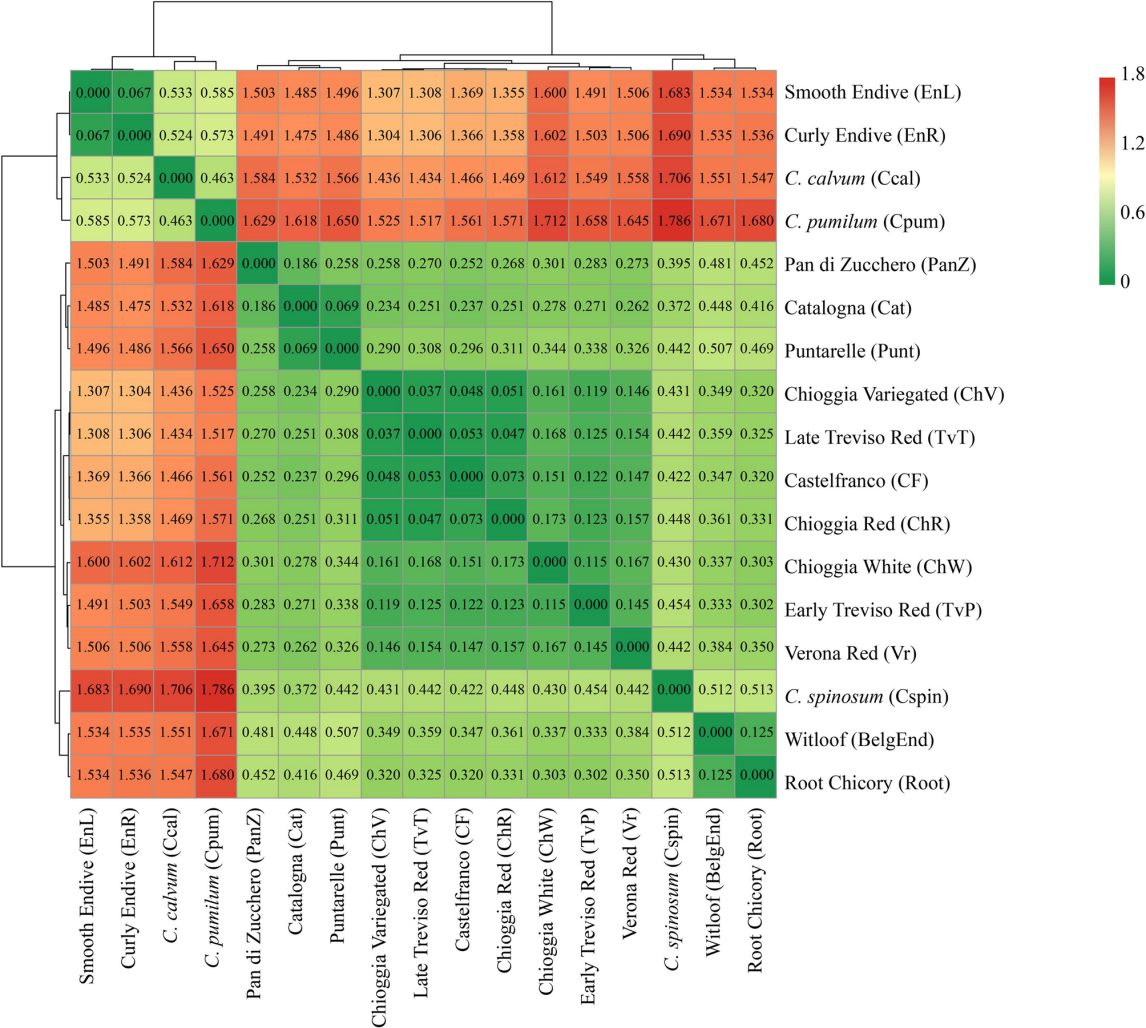
Nei’s average pairwise genetic distance (GD) computed for biotype accessions and wild species

Nei’s genetic distance (GD), computed in all pairwise comparisons (species, botanical varieties, populations and individual samples are detailed in Supplementary Table 3), was plotted as an ordered heatmap for biotypes and wild species (Fig. 4). Generally, the GD among species indicated two main clusters, one comprising the *C. intybus* and *C. spinosum* accessions, while the other comprehending *C. endivia*, *C. calvum* and *C. pumilum*. Notably, GD between the wild and domesticated species was greater in the cluster containing C. endivia (above 0.500) than the one containing C. intybus (0.335) (Supplementary Table 3a). Regarding the GD between the botanical varieties (Supplementary Table 3b), a close relationship was observed between *C. intybus* var. *foliosum* and *C. intybus* var. *sativum* (GD = 0.125). In contrast, the GD between the other *C. intybus* varieties exceeded 0.200. Conversely, the two botanical varieties of *C. endivia* presented the closest relatedness (GD = 0.067). A more detailed investigation at the biotype level (Fig. 4) confirmed the clustering observed in the previous comparisons and further revealed distinctions among biotypes from *C. intybus* var. *latifolium*. Specifically, the radicchio biotype consisted of accessions from TvT, CF, ChV and ChR, with a GD < 0.100, and another consisted of TvP, Vr and ChW, with a GD between 0.100 and 0.200.

To further confirm the GD results, an Fst pairwise comparison analysis was performed in which the accessions were grouped according to their species and botanical varieties. The obtained matrix, reported in Table 4, highlights the strong relationships between botanical varieties from the same species, and the clear distinction between *C. intybus* and *C. endivia*, but with greater genetic variability within chicories than endives. Indeed, although the Fst values within species were less than 0.602 (var. *porphyreum* vs. var. *foliosum*), the Fst values among species ranged from 0.661 to 0.845. From a molecular point of view, the observed values confirmed the high variability observed within *C. intybus* and further highlighted the relationships between the botanical varieties from this species. These results, paired with those from the GD analysis, suggest strong distinctiveness between taxa at all hierarchical levels, which could be exploited in selecting the most informative loci identified.

**Table 4:**
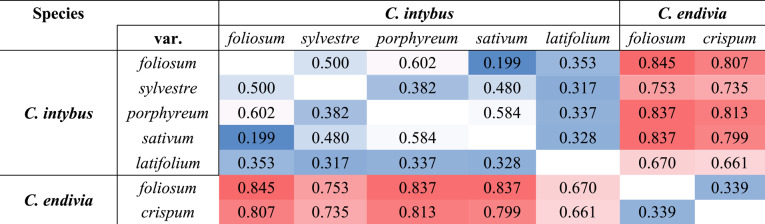
Fst pairwise matrix among botanical varieties and species from the genus *Cichorium*

Based on the GD matrix for single-sample comparison, a UPGMA dendrogram with bootstrap support values was obtained (Supplementary Fig. 1_UPGMA), which separated samples according to their biotype, botanical variety, and species. In a few cases, individuals belonging to different populations of the same biotype clustered together (e.g., TvT1 with TvT2 and TvT3 with TvT4).

**Fig. 5:**
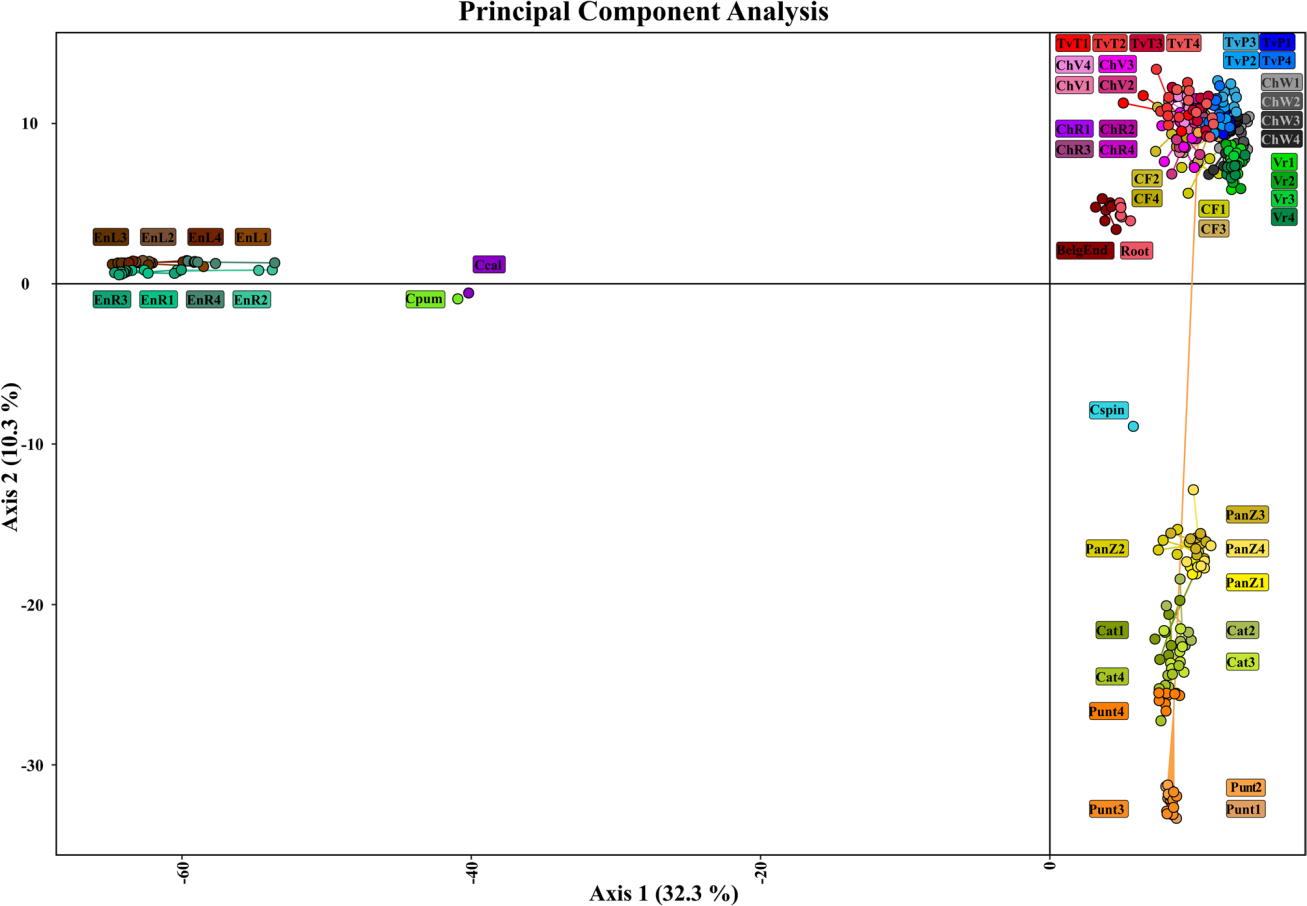
Principal Component Analysis (PCA) computed using 1350 polymorphic loci with 8139 variants identified among the *Cichorium* spp. accessions

Consistent with the findings derived from the UPGMA dendrogram and GD analysis, principal component analysis (PCA) effectively grouped the samples according to their original population and biotype while distinguishing them according to their botanical variety and species (Fig. 5). The first two components of the analysis explained 32.3% and 10.3% of the overall molecular variation, respectively, accounting for a combined 42.6% of the total variation observed in the dataset. The PCA chart revealed that accessions of *C. intybus* and *C. spinosum* were clustered on the right side of the chart, whereas *C. endivia*, *C. calvum* and *C. pumilum* were grouped on the left side. Moreover, the accessions from *C. intybus* were separated into three main clusters according to the botanical varieties considered in this study. Accessions from var. *latifolium* formed a distinct group, with accessions from var. *sativum* (Root) and var. *foliosum* (BelgEnd) positioned nearby but remaining distinguishable. The genotypes from var. *sylvestre* and var. *porphyreum* were also clearly separated and clustered according to their original biotype, with var. *sylvestre* (Cat and Punt) being more distant from the radicchio group than var. *porphyreum* (PanZ). In between the upper and lower groups from the right side of the plot, *C. spinosum* was placed separate from the *C. intybus* accessions but closer to them than the autogamous species. Considering the left side of the chart, accessions from *C. indivia* were grouped close to each other and with low distinctiveness, as were *C. calvum* and *C. pumilum*, which were placed close to each other although separated from the endive samples. This analysis further confirmed the results obtained from the UPGMA dendrogram and confirmed the dataset’s discriminant ability.

Once the discriminability of the analysed accessions was confirmed by the computed genetic statistics and clustering methods adopted, we aimed to investigate the proportion of molecular variability observable among the considered hierarchical groups. Using AMOVA, verified using 999 permutations in the randtest, we examined the molecular variance observable at the four selected grouping levels: species, botanical varieties, biotypes and populations. The findings indicate that the highest grouping level (species) retained the majority of the identifiable molecular variance (55.40%) of the dataset, with the variance decreasing at each subsequent grouping level, with the sample level having an identifiable molecular variance of 1.23%. Moreover, the variance within samples was 18.38%. The randtest supported the results for all grouping levels and further confirmed the strong distinctiveness of the investigated hierarchies. The results of the AMOVA and randtest are reported in Table 5.

**Table 5:** AMOVA and randtest results considering all 5 hierarchical levels: species, botanical varieties, biotypes, populations and samples. Degrees of freedom (Df), summed squares (Sum Sq.), mean squares (Mean Sq.), and respective percentages overall (%) are reported for the AMOVA. The observed variance (Obs), relative standard deviation (Std.Obs), alternative hypothesis (Alter) and acceptance *p*-value (Pvalue) are indicated for the randtest

**Test**	**Df**	**Sum Sq**	**Mean Sq**	**%**
Between Species	4	137516.60	34379.15	55.40
Between Type Within Species	5	73055.66	14611.13	10.98
Between Biotype Within Type	7	45250.28	6464.33	7.32
Between Population Within Botanical varieties	36	49421.99	1372.83	6.70
Between samples Within Population	315	76458.20	242.72	1.23
Within samples	368	78810.11	214.16	18.38
Total	735	460512.85	626.55	100.00

**Random Test (999 permutations)**				
**Test**	**Obs**	**Std.Obs**	**Alter**	*P*value
Variations between Species	645.56	3.16	greater	0.002
Variations between Botanical varieties	127.98	4.66	greater	0.001
Variations between Biotype	85.29	12.08	greater	0.001
Variations between Population	78.03	45.03	greater	0.001
Variations between samples	14.28	5.51	greater	0.001
Variations within samples	214.16	−37.78	less	0.001

### Genetic structure of the core collection and Bayesian clustering

**Fig. 6:**
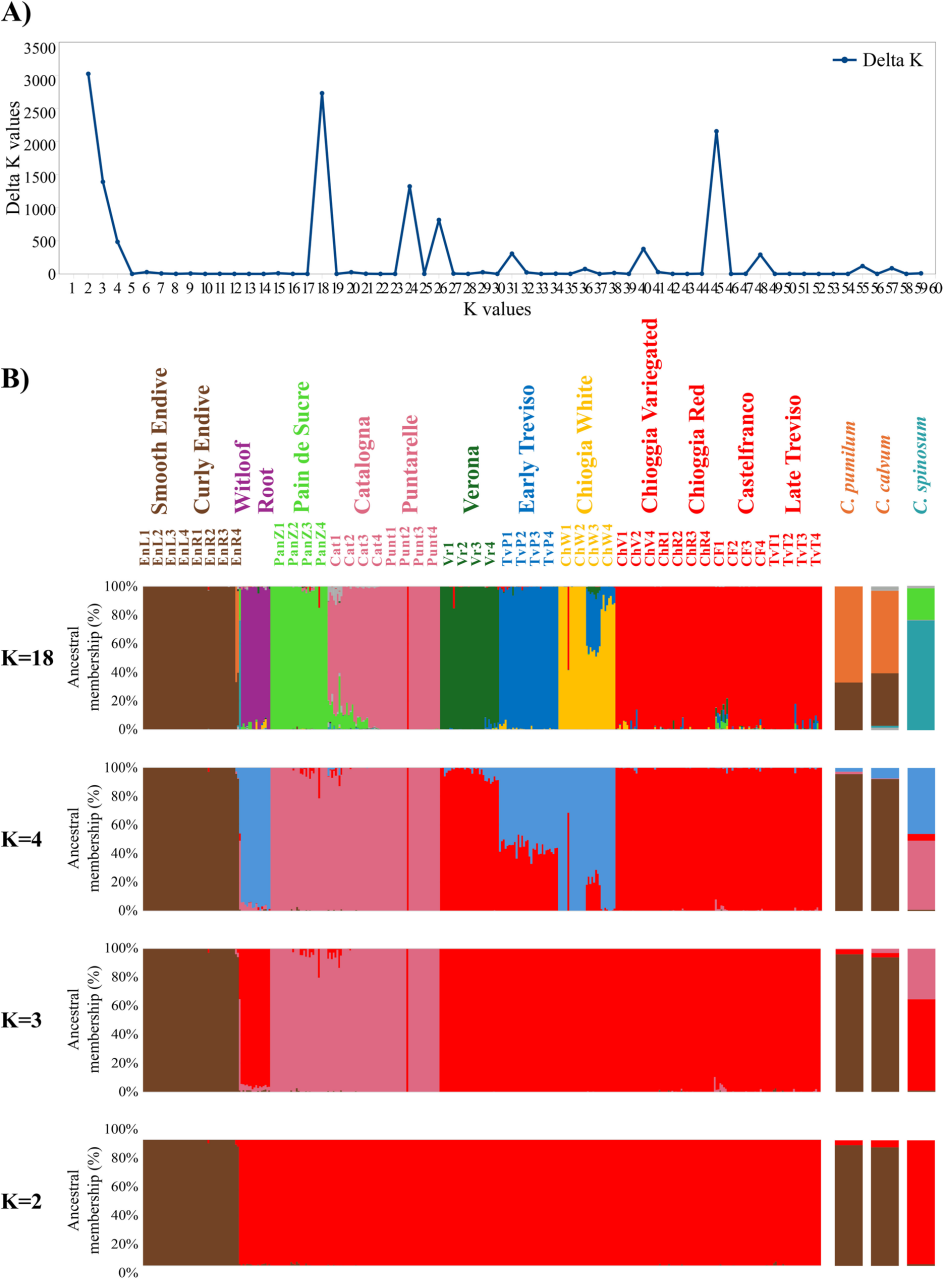
**A** ΔK chart indicating the most likely number of K; **B** Barplots for K = 2, K = 3, K = 4, and K = 18 reporting the membership of each sample to the respective ancestral cluster based on the STRUCTURE results. Biotypes and species names are also reported above histograms to indicate their respective sample/s. Separate histograms are also reported for the wild species described above

The STRUCTURE software was used to reconstruct the ancestral clustering of the analysed genotypes, producing multiple outputs based on ΔK values, which were calculated to identify the most likely number of ancestral clusters (K) for subdividing the core collection and assigning membership to each genotype. As shown in Fig. 6A, the most likely values of K were 2, 3, 4 and 18 based on the ΔK values in the graph. While additional peaks were detected at K = 24, 26, and 45, these higher values were not included in the final analysis due to their reduced biological relevance. Specifically, they resulted in highly fragmented clustering patterns with subclusters showing membership coefficients below 1%, which limited their interpretability and representativeness. Therefore, only K = 2, 3, 4, and 18 were selected for detailed visualization. The STRUCTURE results were plotted as barplot indicating the membership of each sample to the respective ancestral population in all four identified K values (Fig. 6B). For K = 2, the plot highlighted two clusters that divided accessions into endives, *C. pumilum* and *C. calvum* in the first cluster and the remaining species in the second cluster. Furthermore, K = 3 kept endives and the related wild species in one cluster, whereas *C. intybus* var. *porphyreum* and var. *sylvestre* were separated from the other allogamous accession into a third cluster. The result for K = 4 was similar to the previous results but with further separation of *C. intybus* var. *sativum* and var. *foliosum*, which showed complete membership to the newly identified cluster along with three populations of White Chioggia biotypes (ChW1, ChW2, and ChW4) and few admixed samples from ChW3 and those from the early Treviso biotype (TvP). With K = 18, the clustering definition increased and was able to separate accessions into single biotype clusters with almost 100% membership (e.g., PanZ, Punt, Vr, TvP, and ChW), whereas the others remained grouped into major clusters according to their species or botanical variety. Notably, biotypes TvT, CF, ChV and ChR were grouped in a single cluster with a low frequency of admixed accessions (membership to a second group threshold > 10% to define accessions as admixed), whereas the biotype Catalogne (Cat) was associated mainly with Puntarelle (Punt), although it was partially admixed with the cluster containing PanZ. Regarding the wild taxa, for K = 18, *C. calvum* and *C. pumilum* clustered in the same group with more than 50% membership, whereas *C. spinosum* was separated with more than 50% membership into a different cluster.

**Fig. 7:**
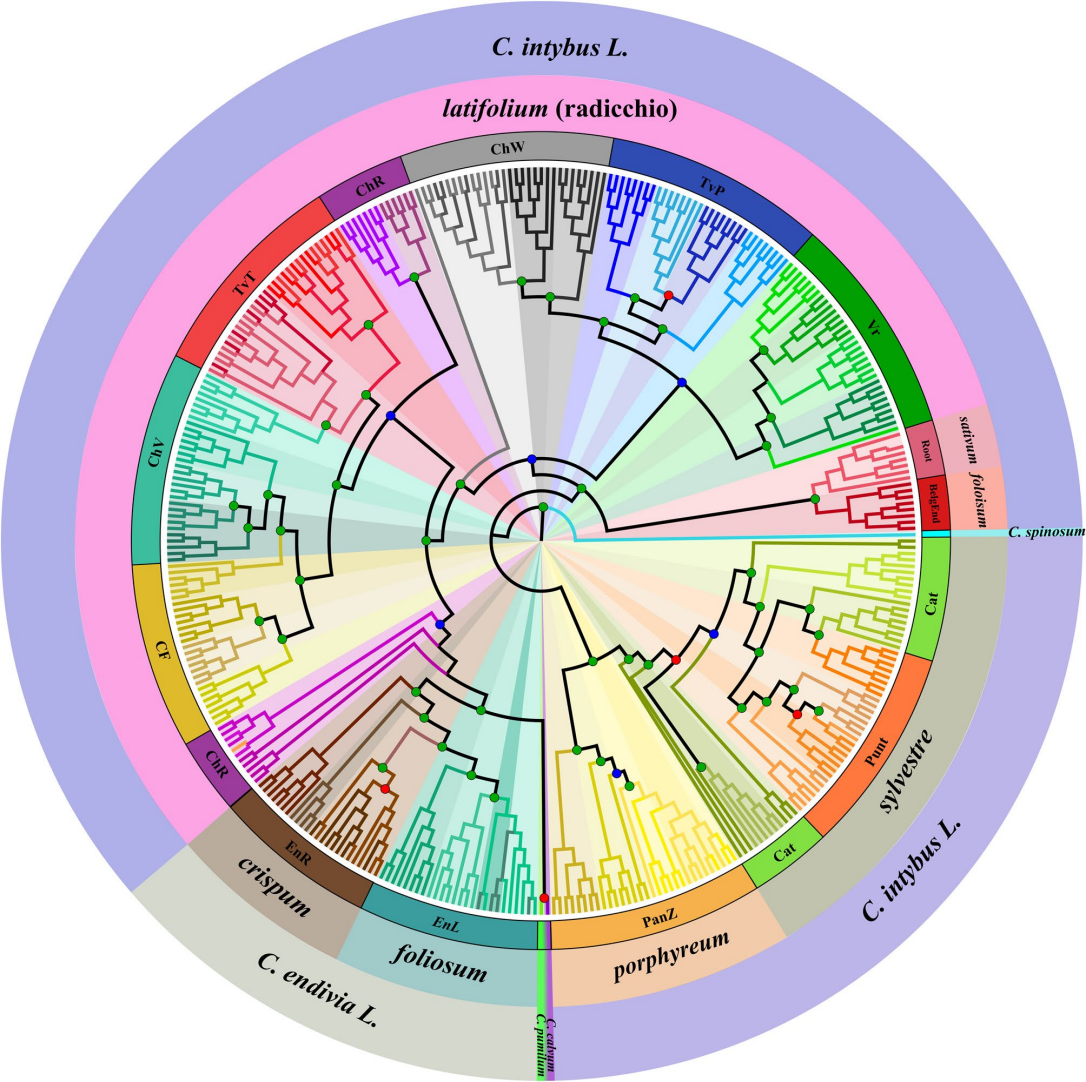
Maximum likelihood cladogram reporting genetic relationships among the accessions of *Cichorium*. Dots in the branches’ nodes indicate supported values for UFB > 95 (in blue ), SH-alRT > 80 (in red ), or both supporting statistics above the thresholds (in green ). Different colours of the terminal arms are used to highlight biotypes and populations (different tones of the same colour are used following the legend provided in Supplementary Fig. 1), whereas arches in the external part of the cladogram indicate botanical varieties and species

After confirming the distinctiveness observable among the taxonomic and varietal groups of the core collection, we aimed to verify the genetic relationships between them. For this purpose, an Maximum Likelihood (ML) analysis was performed, and the results were then plotted as a circular cladogram (Fig. 7). A major grouping of the cladogram separates accessions of the core collection into two main branches agreeing with the results from STRUCTURE software analysis (although based on different computing methodologies), one containing allogamous species (*C. intybus* and *C. spinosum*, with this last used as the root of the cladogram) and the second containing the autogamous ones at the end of the cladogram. Notably, some samples from the ChR biotype were basal to the autogamous species branch, according to previous information about the ancestral genealogy of this biotype which considered this biotype as a possible interspecific derived one between *C. intibus* and *C. endivia* [13, 21], and *C. calvum* and *C. pumilum* samples were equally separated from chicories as endives. Moreover, EnL biotype accessions were placed in the most distant positions of the clade in respect of the allogamous species ones, thus indicating the lower relatedness of this biotype to chicories compared with EnR. Considering the allogamous group, samples were clustered mainly according to their botanical variety (ChR showed exceptions), with var. *porphyreum* basal and var. *sylvestre* residing under one branch and var. *foliosum* and var. *sativum* grouped in a separate clade basal to var. *latifolium* but separated from each other. Regarding the *C. intybus* biotypes, with the exceptions of the ChR and Punt samples, one partially clustered with the radicchios and partially with the endives, and the second formed an inner clade between the Cat samples. The remaining samples were grouped according to their origin, but three subgroups were observed that separated PanZ, Punt and Cat into one branch; Vr, TvP and ChW into the second branch; and TvT, CF, ChV and, ChR into the third branch. In support of these findings, the ultrafast bootstrap (UFB) [53] and the Shimodaira–Hasegawa approximate likelihood ratio test (SH-aLRT) [54] resulted in acceptance thresholds above the main nodes of the cladogram as indicated by the labelled dots in Fig. 7.

## Discussion

The *Cichorium* genus, which belongs to the Asteraceae family and presents two important crops (chicory and endive), is highly diverse regarding its phenotypic and genetic characteristics. Several morphological observations have revealed notable differences in the leaf, flower, and pappus structures of various species from the *Cichorium* genus, which can be useful for phenotype-based species-level discrimination [2, 6, 56]. In the present study, we report the occurrence of trifid stigmas, as a novel floral trait, identified through microscopic analysis and observed in cultivated *Cichorium* species (*C. intybus* and *C. endivia*). Similar morphological variations have been associated with enhanced pollination and seed set in other crops, such as rice [57]. However, to better understand the coexistence of both bifid and trifid stigmas in cultivated *Cichorium* species, further targeted morphological and histological investigations are warranted. Notably, microscopic observations revealed differences in pappus structure between the two varieties of *C. intybus* (var *sativum* and var. *latifolium*), which led to the hypothesis that divergence in pappus structure may also have occurred at the species level. However, resolving the genetic background and phylogenetic relationships within the genus *Cichorium* is considerably more complex than morphological differentiation alone can reveal, especially due to overlapping traits and potential hybridization events among its botanical varieties, biotypes and, in some cases, species.

Despite numerous studies on the phylogenetic relationships within the genus *Cichorium*, challenges remain in fully understanding its genetic structure and relationships among species and their botanical varieties and biotypes. The use of advanced molecular technologies, such as NGS platforms and derived molecular markers, holds promise for obtaining new insights into these complexities and resolving some of the existing ambiguities about the taxonomy of this genus. Current classifications of *Cichorium* species still show inconsistencies, even after several notable studies trying to standardise the nomenclature of these taxa [9–11]. In this study, we aimed to assess the genetic distinctiveness of populations, biotypes, botanical varieties, and species by analysing multiple samples from these taxonomic orders by using multiple statistical approaches applied at different hierarchies. The ddRADseq method implemented on the Ion Torrent platform generated a total of 1.03 billion reads, with over 84% achieving a quality score ≥ Q20, indicating high sequencing accuracy and throughput (Table 2). These metrics support the reliability of the data used for downstream genotypic analyses.

Our study, through the analysis of the obtained final dataset (1350 polymorphic loci in 368 genotypes), allowed the fine-scale analysis of genetic structure, enabling to confirm or revise previous knowledge about genetic relationships within the genus *Chichorium*.

Across all taxonomic levels considered, from populations to species, observed heterozygosity (Ho) was observed to be lower than expected heterozygosity (Hs). This pattern likely reflects the effects of breeding and selection practices on the genetic composition of the accessions, rather than being solely explained by differences in reproductive systems, especially given the presence of both self- and cross-pollinating species. The progressive increase in Hs from the population to the species level indicates greater genetic diversity at broader taxonomic scales, which is further supported by the overall expected heterozygosity (Ht). Nonetheless, Ht highlights the genetic richness still present in these crops, which make them a valuable resource for future breeding efforts. The observed patterns of genetic structure may also serve practical applications, such as plant variety protection (PVP), where clear genetic distinctiveness is essential for the recognition and legal registration of cultivars. Moreover, the Ho comparison between *C. intybus* and *C. endivia* showed the grater variability and tendency to allogamy of the first species, while the second one presented a considerably lower amount of heterozygous loci (Fig. 3). Finally, the Fst values showed strong genetic differences between species, confirming that the adopted dataset was suitable for the molecular characterization and the development of predictive tools in *Cichorium* crops (Table 4). These values also reflect the high genetic diversity within *C. intybus* and help clarify how its botanical varieties are related. Along with the genetic distance results, this analysis highlights distinct separation between groups at all levels, which could be useful for identifying the most informative markers for future applications.

These findings, supported by previous classification results for this genus [11, 21], indicate the strong differences between autogamous and allogamous *Cichorium* species, and further suggest divergently evolved botanical varieties within its species.

Consistent with these metrics, the AMOVA, supported by the randtest results, confirmed the strong differentiation across populations, biotypes, botanical varieties and species of *Cichorium* (Table 5). Notably, the high percentages of molecular variance represented within samples (> 18%), which indicates strong variability within genotypes, was expected due to the allogamous nature of the majority of accessions considered in this study.

When comparing the two major clusters of *Cichorium* species, based on reproductive biology and lifespan, self-compatible species (*C. endivia*, *C. pumilum*, and *C. calvum*) presented a notably greater genetic distance (GD > 0.46) than self-incompatible species (*C. intybus* and *C. spinosum*), with a GD of 0.335 (Fig. 4). This pattern likely reflects not just differences in reproductive strategies, but also the distinct evolutionary paths of these species. Particularly, self-pollinating wild species often experience limited gene flow, which can lead to faster genetic divergence. On the other hand, the self-incompatible group, including both cultivated and weedy types, tend to have more genetic exchange and a shared ancestry, a factor that may explain their lower genetic distances. Within the self-compatible group, *C. endivia* showed very little genetic differentiation between its two botanical varieties (GD = 0.067) (Supplementary Table 3b). The STRUCTURE and ML analyses further reinforced this finding, as both methods were unable to strongly separate these two taxa, thus suggesting significant common ancestry (Figs. 6 and 7).

For the second identified group of species, the genetic proximity between *C. intybus* and *C. spinosum* is supported by previous molecular studies, which struggled to distinguish these two species, even when various molecular markers were used. The close genetic relationship between these two species might suggest a relatively recent divergence that is difficult to identify via a limited number of PCR-based markers. Nonetheless, consistent and heritable morphological differences between *C. intybus* and *C. spinosum* were observed that likely arose from mutations at a few key loci rather than from broad genomic divergence [4]. Notably, *C. spinosum* was found to cluster between the main *C. intybus* group and the leafy biotypes in both the UPGMA and PCA analyses. This pattern suggests a possible shared ancestry or ancestral connection between *C. spinosum* and the leafy forms of *C. intybus*.

*C. intybus* presented notable genetic complexity. Previous studies based on AFLP, ITS, and SSR markers classified *C. intybus* into three main groups: leaf chicory, root chicory, and witloof [4, 11, 16]. More recent research by Barcaccia et al. [13] reported five distinct botanical varieties, which this study also took into consideration. The genetic diversity within *C. intybus* can be attributed to its self-incompatibility, which encourages outcrossing and a broad genetic pool. This diversity is especially relevant for *C. intybus* var. *latifolium*, an agriculturally and economically important variety with numerous biotypes that differ in colour, shape, and agronomic traits. The STRUCTURE analysis (Fig. 6), as well as the UPGMA dendrogram and the ML cladogram (Supplementary Fig. 1 and Fig. 7), distinguished biotypes from var. *latifolium* and clustered them in reduced sub-clusters. Biotypes Vr, ChW, and TvP were closer, although distinct from each other, thus confirming their distinctiveness within *var. latifolium*, whereas TvT, CF, ChV, and ChR formed a single cluster (K = 18 from Fig. 6) or resulted in two major groups (ChR in Fig. 7), suggesting they may share a common genetic background derived from the adopted breeding selection strategies aimed at similar traits or commercial purposes, as previously discussed in Basso et al. [21]. As reported in previous studies, the CF biotype may have originated from the interspecific cross between TvT, or an ancestral *C. intybus* red-leaved wild-type, and *C. endivia* back in the 17th century [20], later being differentiated and selected to fix its present phenotype. However, the clustering observed in this study may reflect the effect of subsequent decades of farmer selection that shaped the phenotypes and genetics of CF and its derived biotypes ChV and ChR.

Further analysis of the phylogeny of the botanical varieties within *C. intybus*, specifically the genetic relationship between *var. foliosum* (witloof chicory) and *var. sativum* (root chicory), revealed a notable genetic distance (GD) of 0.125. This finding supports the hypothesis that witloof chicory, developed in the mid-1800s, originated from the ‘Magdeburg’ root chicory, an ancient cultivar used for producing coffee substitutes [4, 11, 58, 59]. In the UPGMA, PCA and ML tree analyses, these two varieties clustered together but remained distinctly separated, although STRUCTURE analysis did not differentiate but grouped them in a single cluster with almost 100% membership.

In conclusion, our study provides valuable insights into the genomic diversity and phylogenetic relationships within the *Cichorium* genus. Our findings not only support the taxonomic classification of the botanical varieties within *C. intybus* as proposed by Barcaccia et al. [13], but also suggest a potential relationship between var. *sativum* and var. *foliosum* within the broader genus.

Our findings provide novel insights into the genetic diversity within the *Cichorium* genus, which can support the development of molecular tools for the genotypic characterization of the main cultivated species. These tools could be applied in various ways, including the creation of targeted genotyping assays to assist in breeding selection strategies, the development of DUS testing methods for variety registration, and the establishment of efficient traceability assays for cultivated materials and their food derivatives. The high genetic diversity observed within as well as among species can then be adopted to guide conservation initiatives and breeding programs, helping not only to preserve species biodiversity but also to develop more resilient and productive cultivars.

Future research on this genus could focus on understanding how the genetic differences observed in the present research affect the performance and development of individual plants, particularly regarding important agronomic and qualitative traits of commercial interest. However, further research is needed to connect specific phenotypic traits to the genomic regions that control them. Nonetheless, the findings from this study lay a foundation for such future work and for a deeper exploration of the *Cichorium* genus and its genetic resources.

## Supplementary Information

Supplementary Material 1.

Supplementary Material 2.

Supplementary Material 3: Supplementary Table 1. Complete plant material description.

Supplementary Material 4: Supplementary Table 2. Genomic coordinates and haplotypic variants identified after the filtering step. Each row reports a locus retained in the final dataset used for genetic analyses. CHROM: chromosome or contig accession number; Start: the genomic position where the catalog sequence starts; Catalog ID: the identifier assigned to the locus by the Stacks catalog; Polymorphic Sites: positions within the locus that show sequence variation compared to the catalog reference; REF Haplotype (Frequency): the most represented haplotype and its frequency across all samples; ALT Haplotypes (Frequencies): the alternative haplotypes and their respective frequencies.

Supplementary Material 5: Supplementary Table 3. a) Nei’s pairwise genetic distance computed among species in all pairwise comparisons b) Nei’s pairwise genetic distance computed among botanical varieties in all pairwise comparisons; c) Nei’s pairwise genetic distance computed among populations in all pairwise comparisons; d) Nei’s pairwise genetic distance computed among sample in all pairwise comparisons.

Supplementary Material 6: Supplementary Fig. 1. UPGMA dendrogram based on Nei’s genetic distance (GD) computed using 1350 polymorphic loci with 8139 variants among the Cichorium spp. accessions. Red dots on nodes indicate bootstrap support values > 75.

Supplementary Material 7: Supplementary Data 1. FASTA file containing the catalogue of sequences identified by Stacks software.

## Data Availability

Sequencing data of the 368 accessions considered in this study and successfully analysed are available in GeneBank under the project number: PRJNA1190659.

## Abbreviations

AMOVA: Analysis of Molecular Variance
BAM: Binary Alignment Map
BIC: Bayesian Information Criterion
*C. calvum*: *Cichorium calvum*
*C. endivia*: *Cichorium endivia*
*C. intybus*: *Cichorium intybus*
*C. pumilum*: *Cichorium pumilum*
*C. spinosum*: *Cichorium spinosum*
ddRADseq: Double Digest Restriction-site Associated DNA Sequencing
DUS testing: Distinctiveness, Uniformity and Stability testing
Fis: Inbreeding Coefficient
Fst: Fixation Index
GD: Genetic Distance
gDNA: Genomic DNA
Ho: Observed Heterozygosity
Hs: Expected Heterozygosity
Ht: Total Heterozygosity
MAF: Minor Allele Frequency
MCMC: Markov Chain Monte Carlo
ML: Maximum Likelihood
NGS: Next-Generation Sequencing
PCA: Principal Component Analysis
PCR: Polymerase Chain Reaction
PVP: Plant Variety Protection
SH-aLRT: Shimodaira–Hasegawa approximate likelihood ratio test
UFB: Ultra-Fast Bootstrap
UPGMA: Unweighted Pair Group Method with Arithmetic Mean
VCF: Variant Call Format
