# Supplementary figures and images for: A pioneering genotypic and phylogenetic characterisation of Cichorium crops through a genome-scale sequencing for future breeding innovations
Source: BMC Plant Biol. 2025 Jul 3;25:860. doi: 10.1186/s12870-025-06876-1 (PMC12224673; doi:10.1186/s12870-025-06876-1)

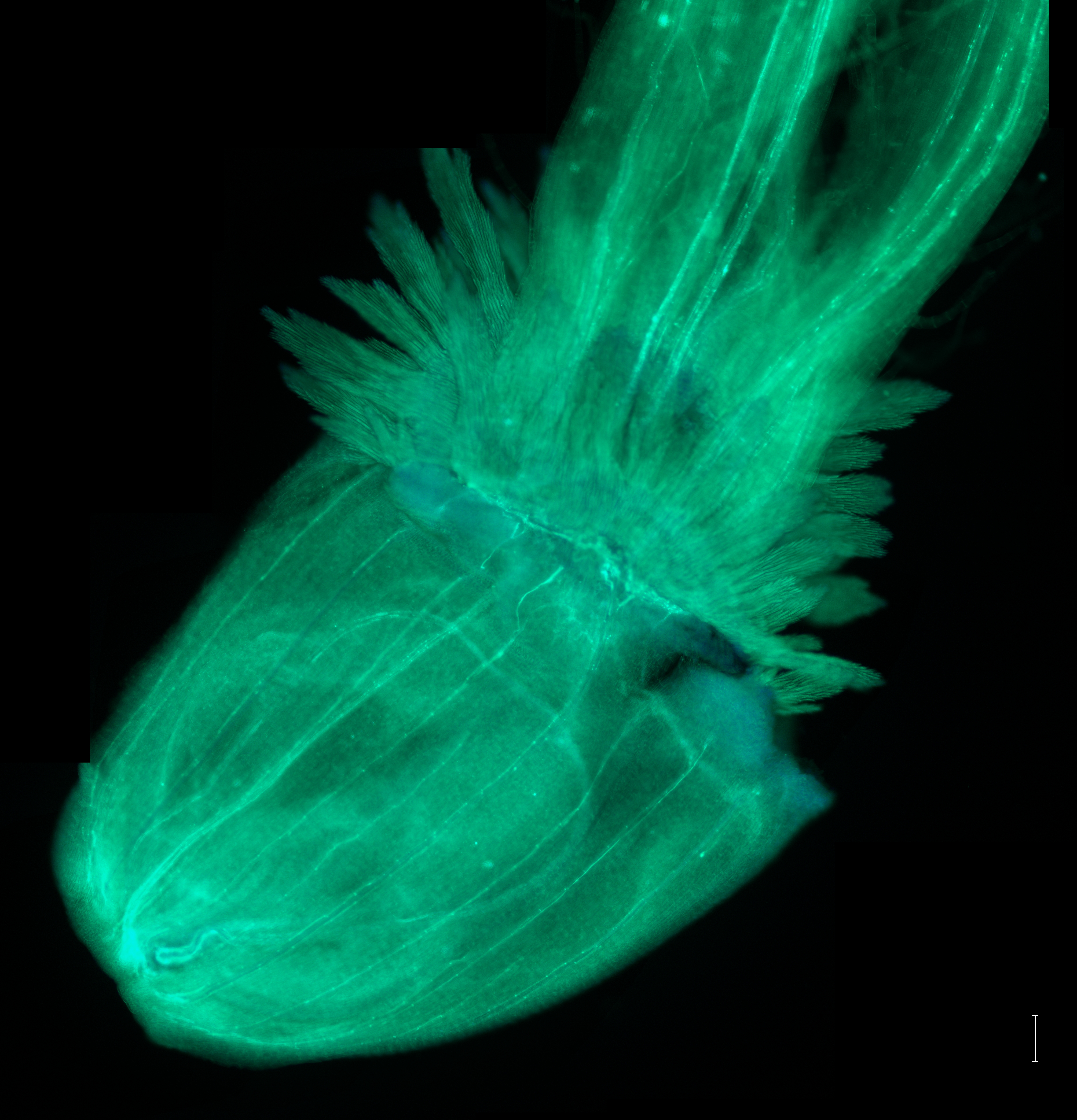

Supplement: Supplementary file 2 — Supplementary Material 2. [file 12870_2025_6876_MOESM2_ESM.zip › Figura 2/2A.jpg]

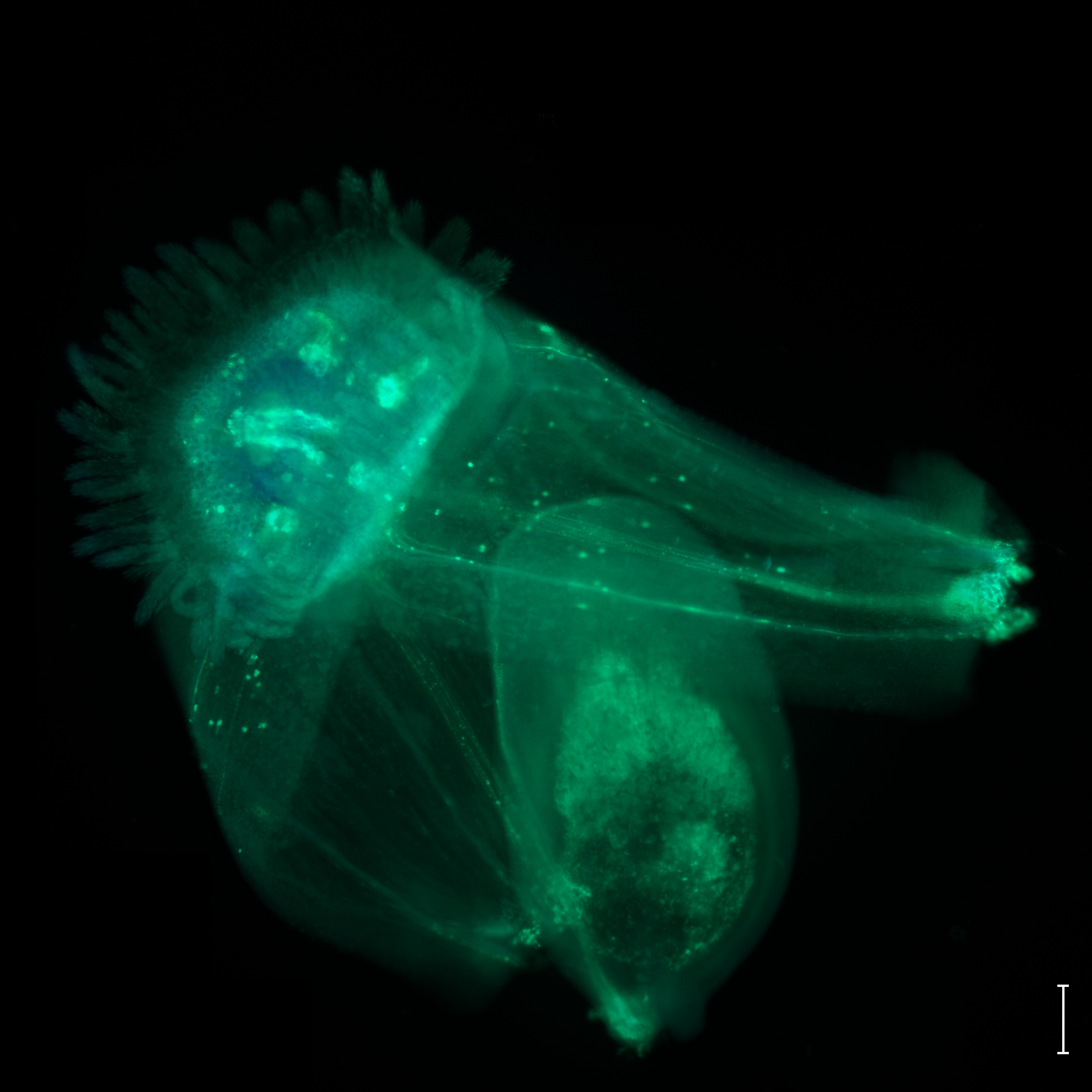

Supplement: Supplementary file 2 — Supplementary Material 2. [file 12870_2025_6876_MOESM2_ESM.zip › Figura 2/2B.jpg]

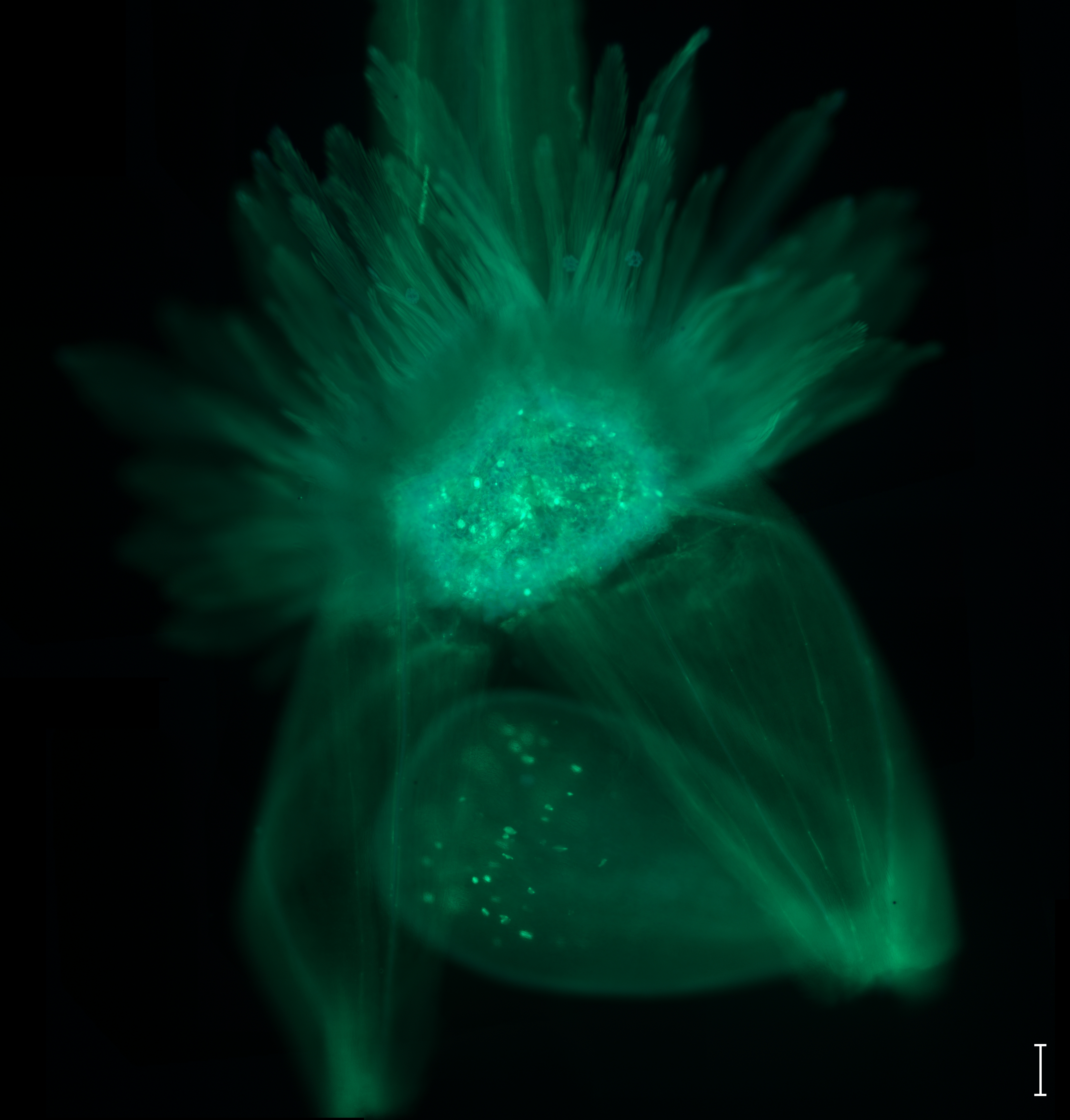

Supplement: Supplementary file 2 — Supplementary Material 2. [file 12870_2025_6876_MOESM2_ESM.zip › Figura 2/2C.jpg]

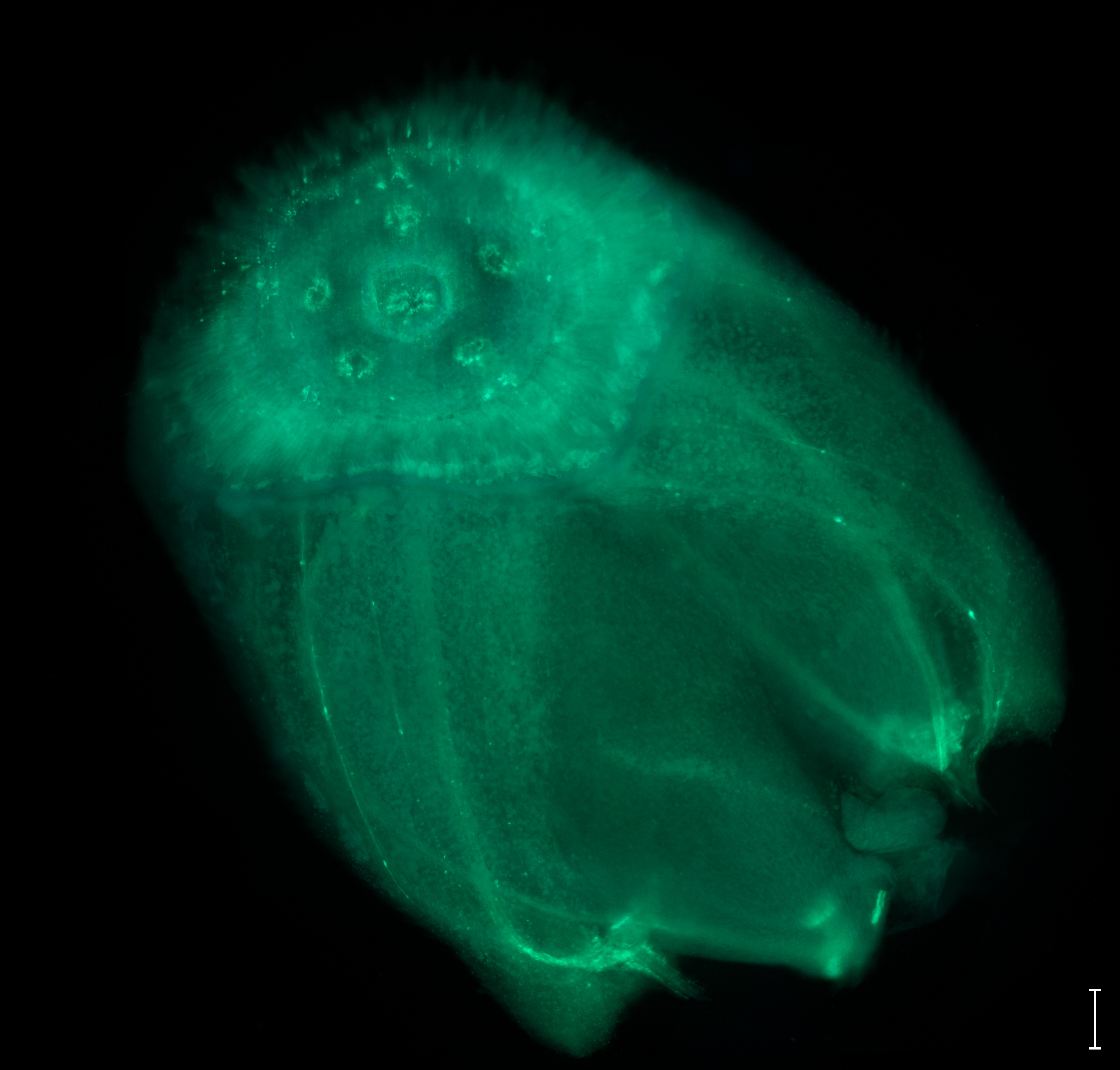

Supplement: Supplementary file 2 — Supplementary Material 2. [file 12870_2025_6876_MOESM2_ESM.zip › Figura 2/2D.jpg]

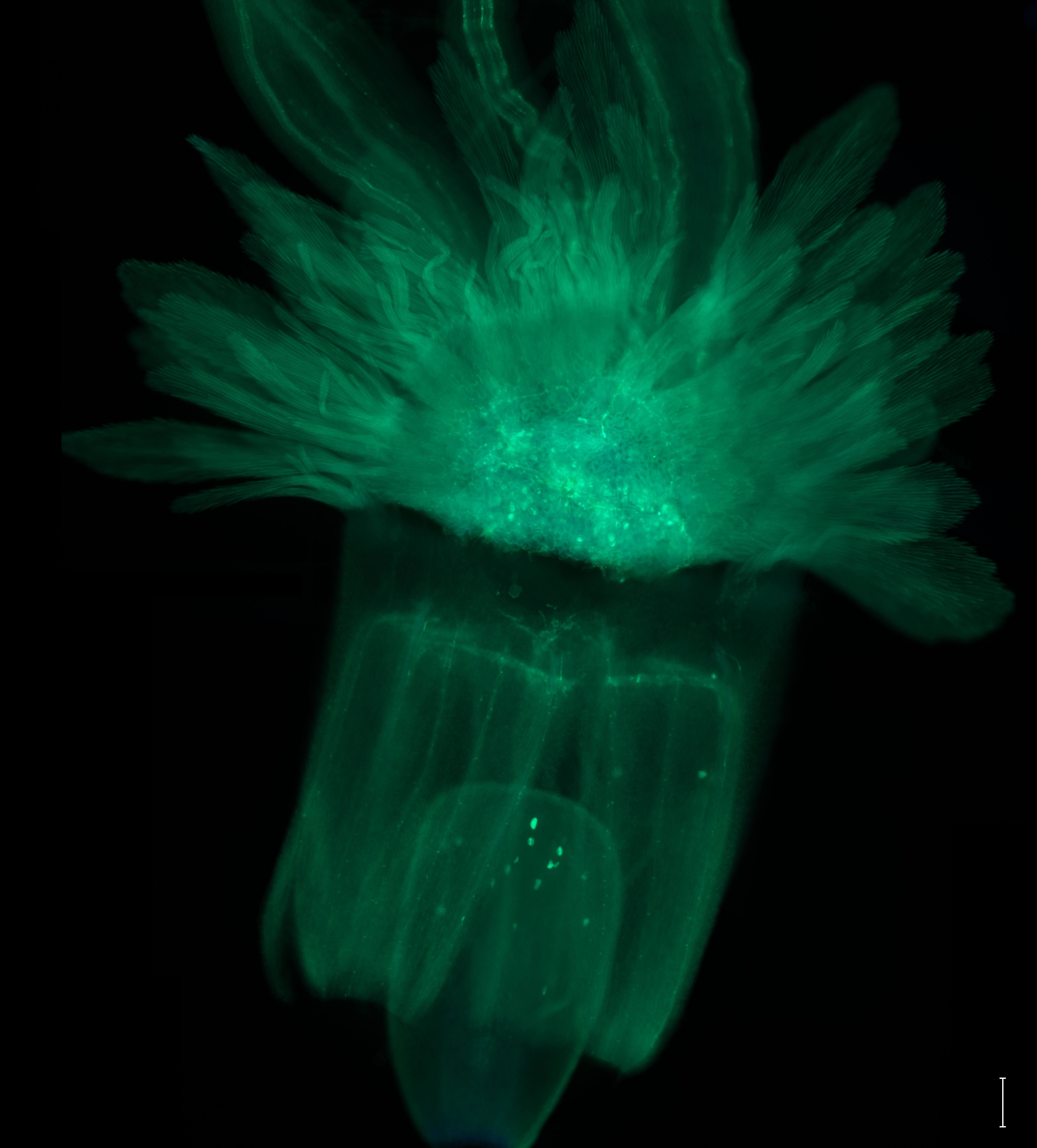

Supplement: Supplementary file 2 — Supplementary Material 2. [file 12870_2025_6876_MOESM2_ESM.zip › Figura 2/2E.jpg]
