# Supplementary material for: A pioneering genotypic and phylogenetic characterisation of Cichorium crops through a genome-scale sequencing for future breeding innovations
Source: BMC Plant Biol. 2025 Jul 3;25:860. doi: 10.1186/s12870-025-06876-1 (PMC12224673; doi:10.1186/s12870-025-06876-1)
Supplement: Supplementary file 6 — Supplementary Material 6: Supplementary Fig. 1. UPGMA dendrogram based on Nei’s genetic distance (GD) computed using 1350 polymorphic loci with 8139 variants among the Cichorium spp. accessions. Red dots on nodes indicate bootstrap support values > 75. [file 12870_2025_6876_MOESM6_ESM.pdf]

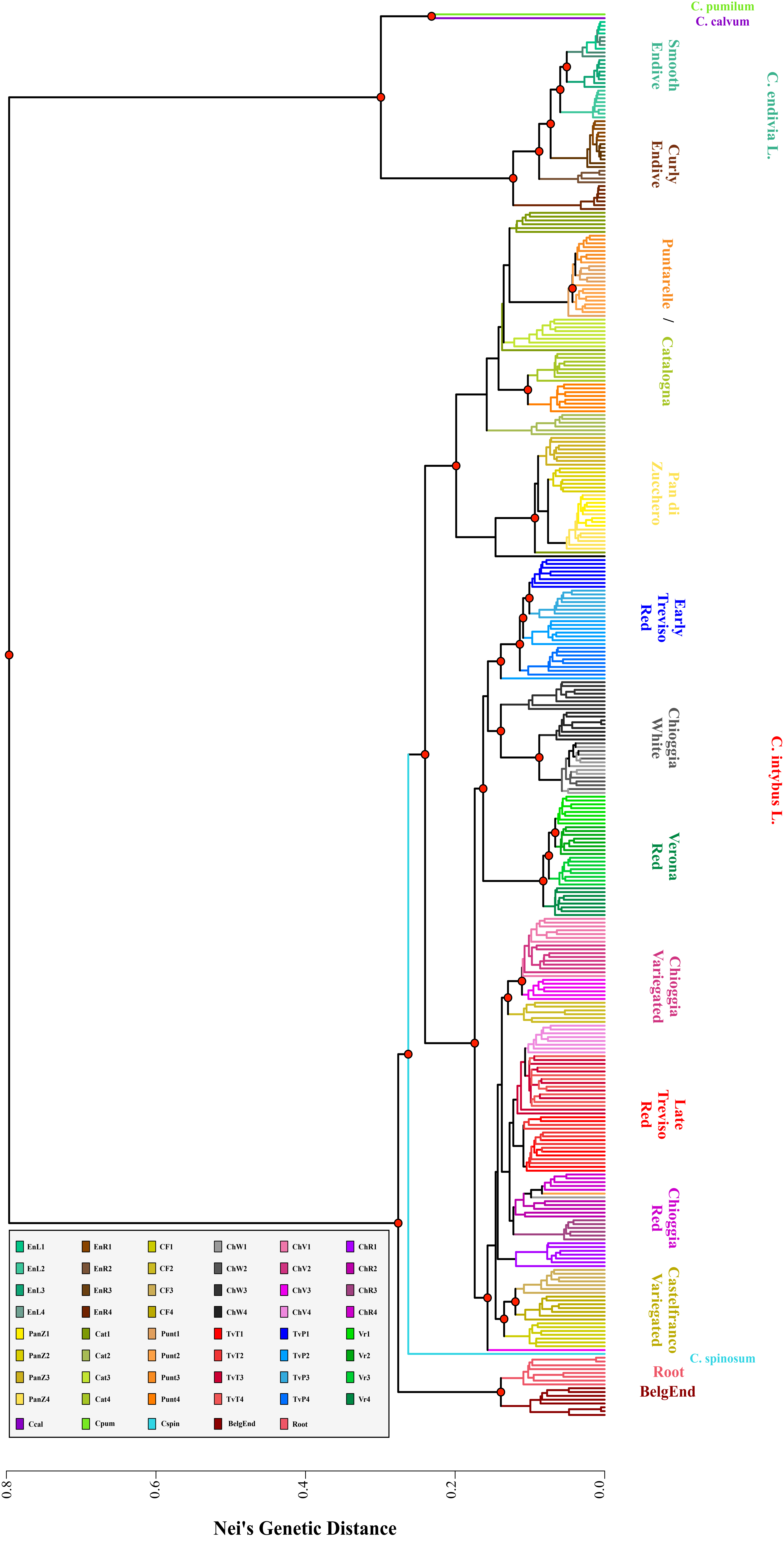

Figure S1. UPGMA dendrogram based on Nei's genetic distance (GD) computed using 1350 polymorphic loci with 8139 variants among the Cichorium spp. accessions. Red dots on nodes indicate bootstrap support values > 75.
